# Novel Noninvasive Paraclinical Study Method for Investigation of Liver Diseases

**DOI:** 10.3390/biomedicines11092449

**Published:** 2023-09-03

**Authors:** Nina Gyorfi, Adrian Robert Gal, Andras Fincsur, Karoly Kalmar-Nagy, Kitti Mintal, Edina Hormay, Attila Miseta, Tamas Tornoczky, Anita Katalin Nemeth, Peter Bogner, Tamas Kiss, Zsuzsanna Helyes, Zoltan Sari, Mihaly Klincsik, Vladimir Tadic, Laszlo Lenard, Andras Vereczkei, Zoltan Karadi, Zoltan Vizvari, Attila Toth

**Affiliations:** 1 Medical and Engineering Multidisciplinary Cellular Bioimpedance Research Group, Szentagothai Research Centre, University of Pecs, Ifjusag Str. 20, H-7624 Pecs, Hungary; 2 Institute of Physiology, Medical School, University of Pecs, Szigeti Str. 12, H-7624 Pecs, Hungary; 3 Department of Medical Biology and Central Electron Microscopic Laboratory, Medical School, University of Pecs, Szigeti Str. 12, H-7624 Pecs, Hungary; 4 Department of Pathology, Medical School, University of Pecs, Szigeti Str. 12, H-7624 Pecs, Hungary; 5 Department of Surgery, Medical School, University of Pecs, Szigeti Str. 12, H-7624 Pecs, Hungary; 6 Department of Laboratory Medicine, Medical School, University of Pecs, Szigeti Str. 12, H-7624 Pecs, Hungary; 7 Department of Medical Imaging, Medical School, University of Pecs, Szigeti Str. 12, H-7624 Pecs, Hungary; 8 Department of Pharmacology and Pharmacotherapy, Medical School, University of Pecs, Szigeti Str. 12, H-7624 Pecs, Hungary; 9 Eötvös Loránd Research Network, Chronic Pain Research Group, University of Pecs, H-7624 Pecs, Hungary; 10 National Laboratory for Drug Research and Development, Magyar Tudósok Krt. 2, H-1117 Budapest, Hungary; 11 Symbolic Methods in Material Analysis and Tomography Research Group, Faculty of Engineering and Information Technology, University of Pecs, Boszorkany Str. 6, H-7624 Pecs, Hungary; 12 Department of Technical Informatics, Faculty of Engineering and Information Technology, University of Pecs, Boszorkany Str. 2, H-7624 Pecs, Hungary; 13 Institute of Information Technology, University of Dunaujvaros, Tancsics M. Str. 1/A, H-2401 Dunaujvaros, Hungary; 14 John von Neumann Faculty of Informatics, University of Obuda, Becsi Str. 96/B, H-1034 Budapest, Hungary; 15 Department of Environmental Engineering, Faculty of Engineering and Information Technology, University of Pecs, Boszorkany Str. 2, H-7624 Pecs, Hungary

**Keywords:** NAFLD, hepatic steatosis, bioelectrical impedance spectroscopy, noninvasive measurement, low-frequency measurement, validation, clinical study, Wistar

## Abstract

Based on a prior university patent, the authors developed a novel type of bioimpedance-based test method to noninvasively detect nonalcoholic fatty liver disease (NAFLD). The development of a new potential NAFLD diagnostic procedure may help to understand the underlying mechanisms between NAFLD and severe liver diseases with a painless and easy-to-use paraclinical examination method, including the additional function to detect even the earlier stages of liver disease. The aim of this study is to present new results and the experiences gathered in relation to NAFLD progress during animal model and human clinical trials.

## 1. Introduction

Nonalcoholic fatty liver disease (NAFLD) is spectrum disease, where more than 5% accumulation of fat buildup appears in the liver in the absence of significant alcohol consumption [1,2]. Spectrum of NALFD ranges from simple steatosis (nonalcoholic fatty liver, NAFL) to steatohepatitis (nonalcoholic steatohepatitis, NASH) with potentially progressive liver fibrosis up to cirrhosis and hepatocellular carcinoma (HCC) [3]. NAFLD is the most common liver disease worldwide, affecting 25–30% of the adult population [4]. The prevalence of fatty liver appears to be highest in men aged between age 40 and 50 and in women aged between 60 and 69 [5,6]. NAFLD can be associated with obesity, metabolic syndrome (MetS), insulin resistance (IR), type 2 diabetes mellitus (T2DM) and cardiovascular diseases [1,2,7,8]. Diet, physical activity and stress level can also modulate the risk of developing NAFLD and the severity of progression [9,10,11]. Nowadays, there is no agreement on an effective pharmacological treatment for NAFLD; therefore, the only currently recommended alternative appears to be lifestyle change [12,13]. The gold standard method to objectively detect the extent of the disease is liver biopsy [14,15]. In addition, laboratory tests and imaging techniques (abdominal ultrasound (US), computer tomography (CT), magnetic resonance imaging (MRI), echo transient elastography (TE)) are used in clinical practice [1,4,16,17,18,19,20,21]. Despite these techniques having shown many advantages, they still have limitations [1,19], such us high cost, ionizing radiation or reduced sensitivity and selectivity in obese patients. Liver biopsy is still considered the reference method for the diagnosis of NAFLD; however, there are several other issues also associated with this method. These include high cost, patient resistance due to potential pain and complications, and the fact that biopsy is an invasive procedure, making it unsuitable as a screening method. Therefore, there is demand to develop a new method that could eliminate these limitations. Examining the electrical properties of tissues could be a promising approach.

The response of biological tissues to electric fields and currents has been intensively studied since the 1970s [22]. Bioelectrical impedance analysis (BIA) is a commonly used body composition analysis technique [23,24]. BIA methods can be divided into single-frequency BIA (SFBIA) and bioelectrical impedance spectroscopy (BIS) [25]. SFBIA measures impedance (Z) at 50 kHz. Since the 50 kHz current does not fully penetrate in the tissue, the SFBIA is not capable for the measurement of the entire sample volume [26]. Conversely, BIS uses frequency series, which can ensure a more representative measurement by testing the lower and higher frequencies at the same time, providing new perspectives for impedance-based research in NAFLD. SFBIA-based detection of hepatic steatosis has been investigated in several clinical studies [27,28,29,30,31,32,33] and small animal models [22,34,35,36]. Despite the great amount of studies on this subject, there is only a limited number of studies available on the assessment of NAFLD using BIS measurement.

Authors have focused on the early detection of NAFLD through a new type of BIS-based analysis. The first step was the development of a proprietary BIS measurement system, followed by the measurement and data collection procedure. A number of technical and methodological solutions with mathematical procedures for data evaluation were developed, which were previously only partially available or not available at all [37,38]. The elimination of residual capacities in drainage was presented by Vizvari et al. [38]. This validation measurement reflected that the frequency range and accuracy are much higher than for state-of-the-art methods. Further, measuring devices that have been developed over the years [39] and early results of clinical trials have been presented previously [40].

The aim of this paper is to investigate whether the BIS-based method can be effectively applied for the detection of hepatic steatosis and the progression of liver disease severity.

## 2. Materials and Methods

### 2.1. Bioimpedance Spectral Measurement

Accurate and reliable bioimpedance spectrum (BIS) measurement is an essential requirement for drawing relevant conclusions for biological processes. Even with the most advanced methods developed for this purpose, the accuracy and efficiency of the impedance measurements in biological systems, and therefore the interpretability of biological data, is limited by the systematic occurrence of measurement error parameters such as residual impedance, parasitic capacitance, generator anomalies, etc. Authors have mainly specialized in low-frequency (<10 Hz) BIS measurements [37,38]. Vizvari et al. [38] proposed a method that is capable of rejecting anomalies while providing simpler data collection for measurements in the ultralow frequency range. Using the proposed impedance measurement technique, the characterization of biological systems can be performed more accurately [38].

#### 2.1.1. BIS Measurement Principal

**Figure 1:**
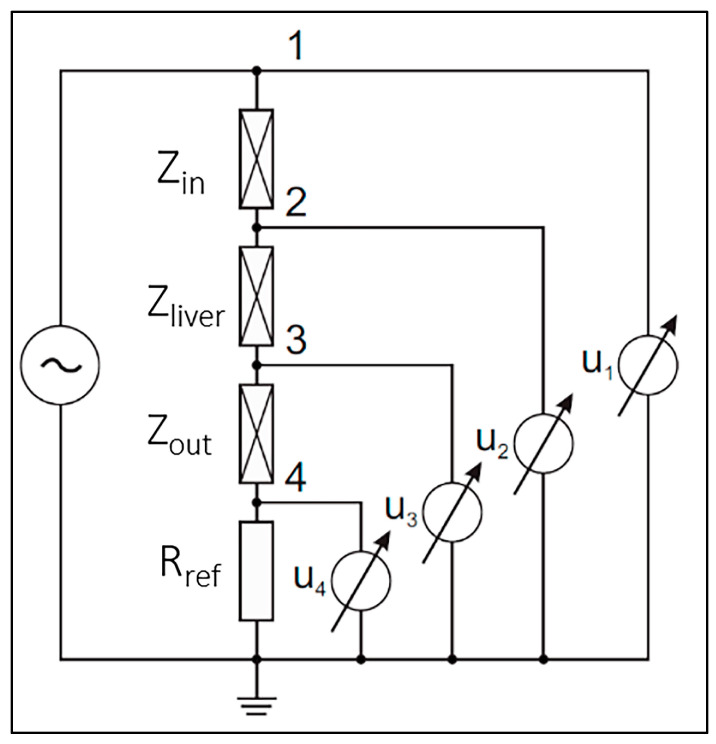
BIS measurement principle.

BIS measurements were carried out using a self-developed four-electrode measurement and data collection procedure published by Vizvari et al. [38]. The main principle of the self-developed BIS measuring instrument is illustrated in Figure 1. The purpose of the measurement is to define the Z_liver_ impedance values at multiple frequency points. Z_in_ and Z_out_ represent the contact impedance corresponding to an adhesive body surface electrode and the body. Although, the contact impedances do not appear in the Z_liver_ impedance value. The Z_in_ and Z_out_ values suppress the Z_liver_ impedance due to the behavior of the model at low frequencies. Therefore, it does not appear in the resulting impedance (Z) of the model. The effectiveness of the method is based on the use of a reference resistance (R_eef_) against the unknown impedance (Z_liver_) and compared. A voltage generator is used to excite the measured object, then the voltage values u_1_, u_2_, u_3_ and u_4_ are measured in nodes 1, 2, 3 and 4. During the data collection procedure, the grounding point (0 V) of the system is connected directly to the tested material and the measured potential values are compared to this point. The potentials measured in nodes 1, 2, 3 and 4 are subtracted from each other to eliminate errors in the measured data.

The measured raw impedance spectrum data are evaluated using the following equation [38]:

$$Z=R_{ref}\frac{u_{2}-u_{3}}{u_{4}}$$

#### 2.1.2. Small Laboratory Animal Model BIS Measurements

**Figure 2:**
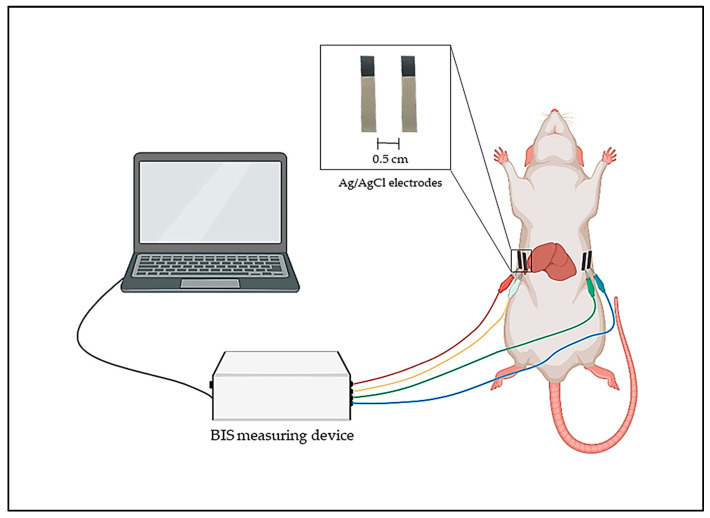
The schematic of the self-developed animal model BIS measurement principle.

The BIS measurements were performed using intraperitoneal anesthesia with a mixture of ketamine and xylazine (ketamine 100 mg/kg and xylazine 10 mg/kg). The dose of the mixture was 0.15 mL/100 g body weight. Fur was removed from the abdominal regions. Ag/AgCl self-adhesive skin surface short electrode pads (Bodystat Ltd., Isle of Man, UK) were used, same as is applied in human BIS measurement; however, the surface area of the electrode was reduced to a quarter. The electrodes were applied to the level of the lower rib arches on both sides of the body, according to the human BIS protocol. The outer electrodes were placed on both sides of the body, and the inner electrodes were placed approximately 0.5 cm away from them (Figure 2).

Figure 2 shows the assembled measuring system. This methodology was previously validated [38]. The silver box represents the self-developed BIS measuring instrument equipped with different colored cables (red, yellow, green, blue). The generator is connected with red and blue wires, while the measuring channels are connected with yellow, green and blue wires. The cables end with crocodile clips, which are connected to the self-adhesive body surface electrodes. The BIS measuring device is connected to a computer, where the measurement results are displayed during the procedure. The data collection process can start when the cables have been connected to the electrodes in the appropriate order.

**Figure 3:**
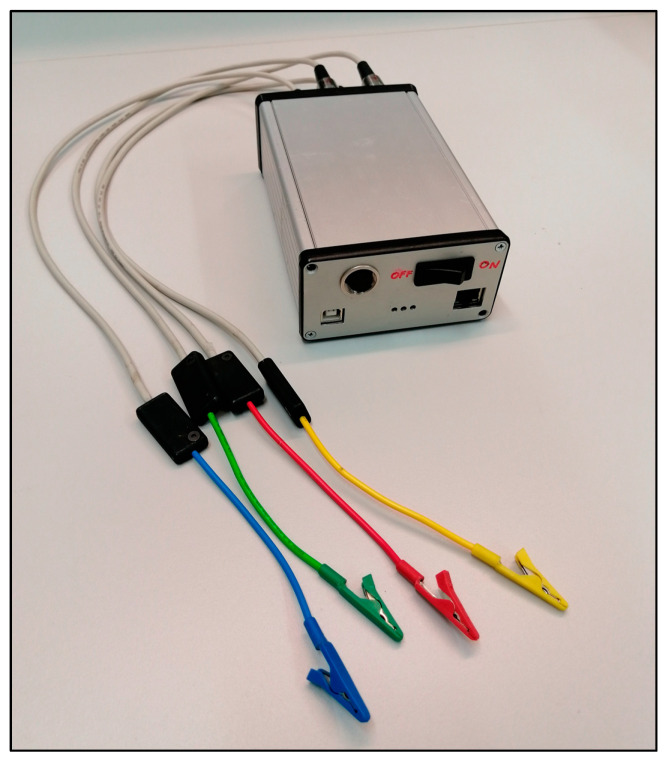
The BIS device used for small animal experiment.

The technical parameters of the prototype (Figure 3) are the follows:

- Generator signal: monochrome sine;
- Voltage generator amplitude: 1 mV–1 V (peak to peak);
- Frequency range: 1 Hz–100 kHz;
- Number of measuring channels: 4;
- Stored measurement result: Transfer function (absolute value and phase);
- Dynamic range: 140 dB;
- Measurement accuracy: <1 ppm magnitude and <±0.001° phase;
- Resolution of measurement result: double precision (fixed point arithmetic);
- Input AD conversion, generator DA conversion: 24 bits;
- Measurement function: bioimpedance spectrum measurement;
- Power supply: Battery operated.

The measurement was carried out with 1 V peak-to-peak voltage. Frequency sweeping was conducted in a frequency range of 1–100 kHz, measured at 5 points per decade. Five measurement points were set per decade, resulting in 26-point spectrum data. Five parallel measurements were made to eliminate interference during measurement by averaging.

#### 2.1.3. Clinical BIS Measurements

**Figure 4:**
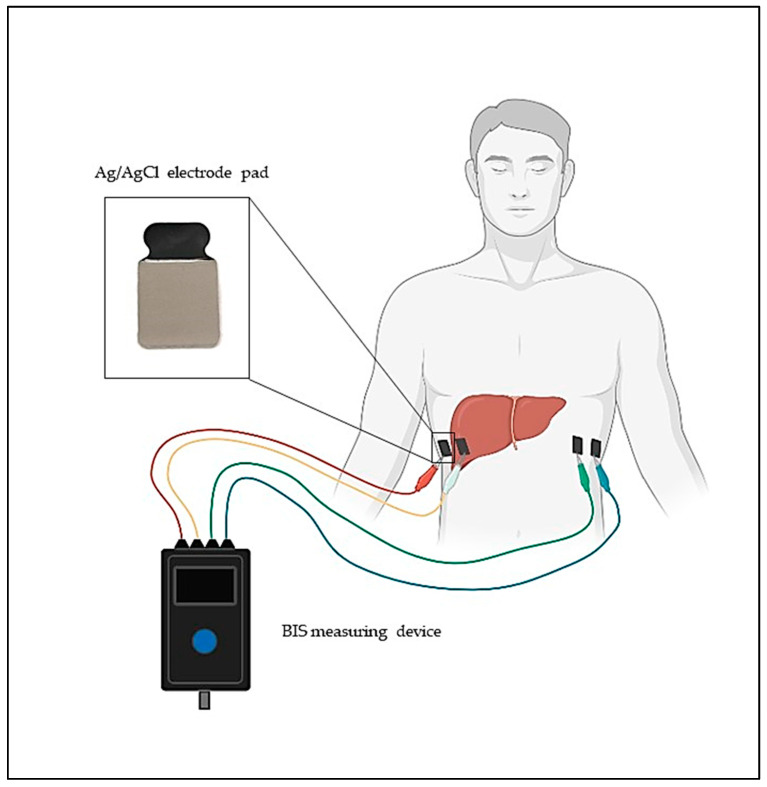
The schematic of the self-developed clinical BIS measurement principle.

The first step in the clinical measurements was to place the patient in a comfortable sitting or lying position. The Ag/AgCl skin surface adhesive electrodes (Bodystat Ltd., Isle of Man, UK) were placed at the level of the lower rib arches on both sides of the body, as the liver can be examined at this cross-section with the greatest extension. Furthermore, measuring the cross-section only examines the area around the liver; therefore, the measured data are not affected by the total body composition values. The outer electrodes were placed on both sides of the body and the inner electrodes were placed approximately 5 cm away from them (Figure 4). After the cables are connected to the surface electrodes, the instrument is ready for automatic data collection.

**Figure 5:**
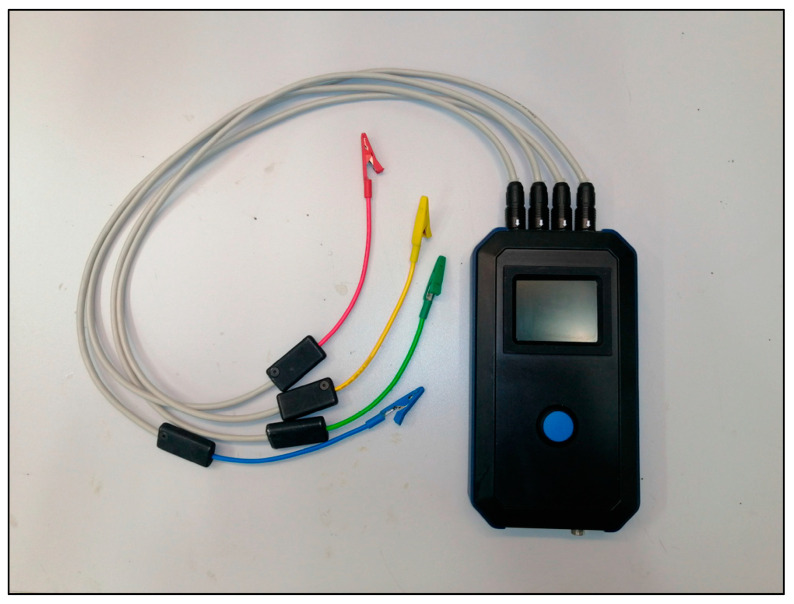
The BIS device used for clinical measurements.

The authors developed several BIS instruments for the detection of NAFLD with the same technical parameters and measurement protocol [39]. The clinical BIS measurements were carried out using the latest self-developed BIS device, as shown in Figure 5. The new miniaturized size allows for the easy mobilization of the instrument in the clinical environment. Since the device can be operated without a computer, it takes only minimal space and can be placed directly next to the clinical bedside. Data collection is automatically performed via the connected USB device, requiring no special skills, by the user. Due to the simple use and compact size, the latest generation BIS instrument can be routinely applied in clinical practice for the investigation of NAFLD.

Data were collected using 1 V (peak-to-peak) excitation signals over a wide frequency range, from 1 Hz to 100 kHz. Five measurement points were set per decade, resulting in 26-point spectral data. Five parallel measurements were made to eliminate interference during measurement by averaging.

### 2.2. Animal Experiment

To establish a new potential diagnostic method for NAFLD, a translational, reproducible animal model is essential. Several rodent models have been used to observe the clinical features of the human NAFLD [41,42]. These models can accurately summarize the histopathological features of the disease under appropriate dietary conditions.

Diet, nutritional patterns and different types of high-calorie nutrients are important regulators and contributors to many diseases, including the development, progression and management of NAFLD. The animal models of NAFLD are mainly based on different types of diets, such as the high fat, fructose and glucose diet that was applied in the present case. High levels of fructose and glucose are a common feature of Western-style diets and are likely to result not only in the development of NAFLD, but also trigger insulin resistance.

Two different experiments were conducted. In the first experiment, a liquid-based intragastric tube feeding was applied. In the second experiment, a solid high-fat rodent supplement was provided, as well as fructose and glucose being added to the drinking water. The intragastric polyethylene feeding tubes were inserted into the antrum of the stomach with a surgical procedure under intraperitoneally administered anesthesia. The anesthetic was a mixture of ketamine and xylazine (ketamine 100 mg/kg and xylazine 10 mg/kg), dosage of the mixture was 0.15 mL/100 g body weight. The postoperative reconvalescence period lasted 4–6 days. The dietary phases lasted for three months.

This research was approved by the Ethical Committee for Animal Welfare of the University of Pécs and the National Scientific Ethical Committee on Animal Experiments (license number: BA02/2000-3/2018).

#### 2.2.1. Animals

For the NAFLD animal model experiment, 60 male Wistar rats with a body weight of about 300 g were used. The animals were kept in controlled environmental conditions (22–24 °C and 12 h light/dark cycle) for at least 3 weeks before the experiment, where the rats were conditioned to human hands (handling). During this time, water and rodent chow were provided ad libitum. Their body weight was measured twice a week. The animals were randomly divided into 3 groups: 70% high-fat diet, 35% high-fat diet and control group, where each group contained 10 animals.

The experiment was conducted two times with the same groups, with the difference that in the first case, only intragastric feeding with a high-fat milk-based dietary fluid was provided; however, in the second experiment, the animals consumed only solid food and fructose/glucose mix was added into their drinking water.

#### 2.2.2. Diet

After the postoperative phase, the dietary phase was started, including a daily liquid high-fat dairy-based diet. An 8 mL/20 g body weight of dietary fluid was injected daily using a Cole–Parmer syringe infusion pump device. A high-fat milk-based dietary liquid was prepared with 20 g fat/100 mL (70% high-fat diet) and 10 g fat/100 mL (35% high-fat diet) milk-based liquid (ex Nutridrink). The fat content of the 70% high-fat dietary fluid consisted of 19 g butter oil and 1 g soybean oil to provide essential fatty acid intake, with a caloric value of 19.34 kJ/g, where 7.74 kJ/g was provided by the fat content. The 70% high-fat liquid diet components according to their energetical value were allocated in the following order: 70% energy from fat, 11% energy from carbohydrate and 18% energy from protein. According to their energy values, the ingredients of the 35% fat liquid diet consisted of 35% energy from fat, 41% from carbohydrates and 24% from protein. The milk-based liquid was placed in a 10 mL syringe and mounted on the pump device. The gastric tube was washed with physiological salt solution previously and after the injection to prevent clogging.

In the second experiment, the treated groups were fed per os with C 1090–30 and C 1090–70 special high-fat solid rodent diets (Altromin, Lage, Germany) daily; furthermore, 23.1 g/L of fructose and a 18.9 g/L of glucose were added into the drinking water to induce an imbalance in liver fat and carbohydrate metabolism.

The dietary phases lasted for three months. In both experiments, there was a control group where water and standard rodent chow (Altromin, Lage, Germany) were provided ad libitum.

#### 2.2.3. Supplementary Examinations

Body weight was measured twice a week throughout the dietary period. In order to evaluate the results provided using BIS analysis, contrast-enhanced abdominal computed tomography (SkyScan 1176 Micro-CT) was performed after the dietary period was completed. The analyses were performed at the Szentágothai János Research Centre of the University of Pécs. The anthropometric values (body weight, body length, abdominal circumferences) of the rats were measured under intraperitoneal anesthesia before the BIS measurement. Afterwards, blood samples were collected from the left ventricle of the heart into sterile blood slides to determine the levels of liver function enzymes (ALAT, ASAT), cholesterol and triglycerides.

In the end, the liver was removed for further pathological examination after the overdosage of the given anesthetic was performed. The samples were immediately stored in paraformaldehyde. The tissue samples were evaluated to determine the degree of steatosis according to the Bedossa SAF (steatosis (S) + activity (A) + fibrosis (F)) score system.

### 2.3. Clinical Study

#### 2.3.1. Patients

This study involved more than 50 patients. The important criteria for patient selection was that the patients should undergo abdominal operations where liver biopsy could be performed relatively simply without significantly increasing the risk of the surgery. As a further consideration, the authors identified the inclusion of a heterogeneous group of patients with different stages of NAFLD, ranging them from a healthy liver to advanced steatosis. Therefore, patients who were undergoing gall bladder removal surgery (cholecystectomy) were found to meet these two criteria most effectively.

All patients underwent BIS measurement a day before surgery and liver tissue sampling during surgery. By examining patients who had undergone tissue sampling, it was possible to compare their histological results with the bioimpedance data.

The ethical clearance for this study was obtained under the study “Investigation of the peripheral and central regulation of metabolism using bioimpedance measurements, human behavioural and complex neurophysiological methods” from the Regional and Local Research Ethics Committee of the Clinical Center, University of Pécs, case number 6752/2018. The written informed consent was obtained from all study subjects in the local language.

#### 2.3.2. Statistical Data

Statistical data collected during the measurement included the patient’s sex, age, weight, height and waist circumference. Body mass index (BMI) has been calculated as body weight (kg) divided by height (meters) squared. Body mass index can be classified into normal (BMI 18.5–24.9), overweight (BMI 25–29.9), mildly obese (BMI 30–34.9), moderately obese (BMI 35–39.9) and severely obese (BMI > 40) categories [43].

#### 2.3.3. Histological Examination of the Liver Sample

Liver histological examination is still necessary, since biopsy remains the gold standard for the diagnosis of liver diseases and it is the only reliable method that allows for the differentiation of NAFLD from NASH. The biopsy samples were evaluated according to the internationally accepted SAF scale values. This scoring system separately evaluates the degree of steatosis (S0–S3, based on the amount of steatosis), the degree of activity (A0–A4, by adding degrees of ballooning and lobular inflammation each from 0 to 2) and the stage of fibrosis (F0–F4).

This paper focuses exclusively on the degree of steatosis while eliminating the changes caused by other values of the SAF score. The amount of liver fat associated with the different stages of steatosis is shown in Table 1.

### 2.4. BIS Data Evaluation

During the BIS measurements, the Z impedance of the tested material spectrum data was calculated at each frequency point using the potential values u_2_, u_3_ and u_4_ according to the equation presented in Section 2.1.1 [38].

The measured Z spectra are displayed in a lin–log diagram, where the x-axis represents the frequency in Hz and the y-axis is the magnitude in ohms.

## 3. Results

This section presents the results obtained from the animal experiments, as well as from the clinical trials.

### 3.1. Animal Experiment

#### 3.1.1. BIS Measurements

The BIS measurements were performed under intraperitoneal anesthesia at the end of the 3-month high-fat dietary period. The measurement results were validated using SAF-score based evaluation of liver histology samples.

**Figure 6:**
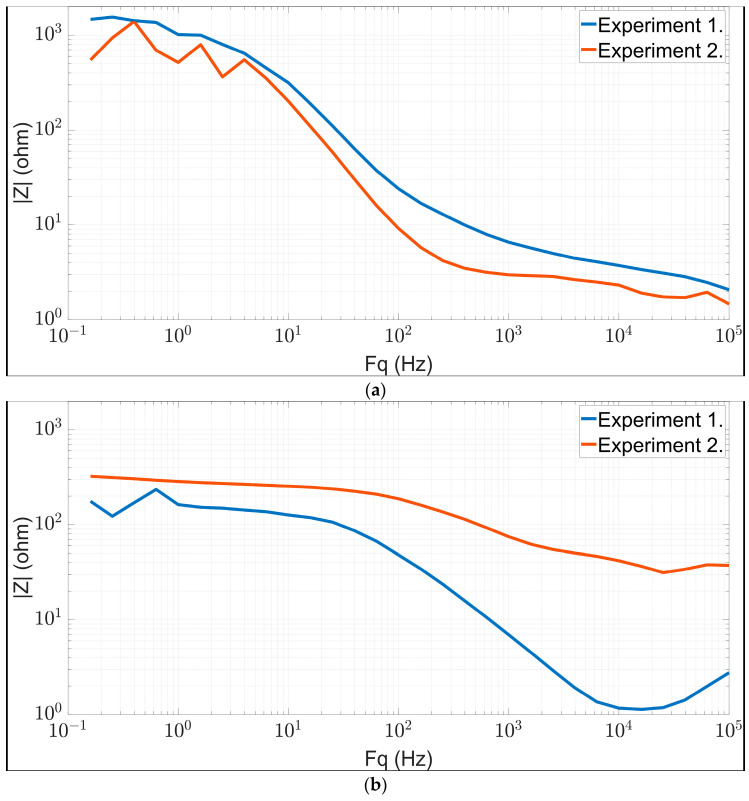

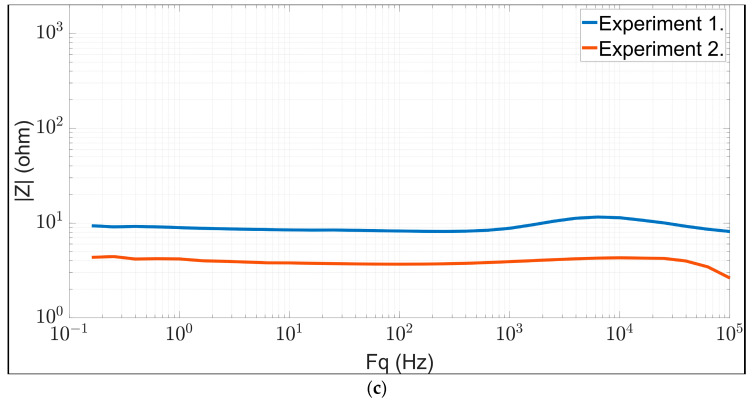
BIS curves in Wistar rats grouped by SAF score obtained from histopathology: (**a**) SAF 0+0+0, (**b**) SAF 1+0+0 and (**c**) SAF 2+0+0. The blue-colored curves represent the animal from the first experiment, and the red-colored curves represent the animal from the second experiment.

Figure 6 shows the most typical BIS spectra measured in rats after a 3-month high-fat diet period. Based on the histological results, the BIS results were classified into three different groups. Figure 6a shows the results of animals with a SAF score of 0+0+0, (b) 1+0+0 and (c) 2+0+0 based on the liver histological samples. The evaluation criteria are the same as presented in the human clinical study (Section 2.3.3). Figure 6 shows the measured spectral data of two animals per graph, where the blue-colored curves represent animals from the first (intragastric, high-fat liquid-based feeding) and the red-colored curves represent animals from the second experiment (per os, high-fat solid food + glucose/fructose). The measured Z spectra are plotted in a lin–log diagram, where the x-axis represents frequency in Hz and the y-axis represents magnitude in ohms.

Figure 6a shows BIS curves of animals with SAF score results of 0+0+0, meaning that that all the values of steatosis, activity and fibrosis were zero, based on the liver histological sampling results. This group shows the BIS curves of the animals from the control group fed with normal rodent chow during the 3 months of the dietary experiment. The BIS curves from both the first and second experiment show a monotonically decreasing trend with a breaking point of around 100 Hz. After the breakpoint, a moderate downward phase was followed.

Figure 6b shows the BIS spectra of animals that were on a high-fat diet with 1+0+0 SAF score result. Here, the results of two different animals show a larger variance. Initially, the blue curve indicates a plateau phase from low frequency to about 100 Hz, followed by a sharply monotonic decreasing phase up to 1000 Hz, finally ending with an upward trend in the recorded impedance values. The red curve takes on decreasing impedance values after a corresponding straight plateau phase from a breakpoint around 100 Hz.

Figure 6c shows the BIS spectrum of high-fat diet animals with 2+0+0 SAF results. In this group, the BIS values in both cases were constant over nearly the entire frequency spectrum.

#### 3.1.2. Statistical Data

Table 2 summarizes the physical characteristics of Wistar rats in the animal experiment. Data were expressed as the mean and standard deviation of the body and liver weight and the abdominal circumference. The majority of participants can be classified as animals without steatosis (SAF 0+0+0) and with mild steatosis (SAF 1+0+0). The reason for the high number in the first group is due to the absence of steatosis in the control group. The initial body weight was about the same for all animals. The average weight increase at the end of the 3-month diet period, and it was the lowest in the case of the first group (83.4 g), while the second and third groups showed remarkable increases (206.1 g and 229.8 g). An increase in liver weight was observed in the second and third groups compared to the first group. In line with the trends in animal weight, abdominal circumference was the lowest in the SAF 0+0+0 group and the highest in the SAF 2+0+0 group.

#### 3.1.3. Micro-CT Scan

**Figure 7:**
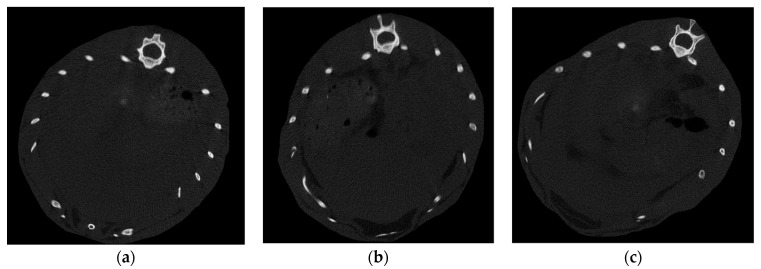
Microcomputed tomography (micro-CT) scans of Wistar rats: (**a**) SAF 0+0+0 (control animal) group, (**b**) SAF 1+0+0 group (35% high-fat diet animal) and (**c**) SAF 2+0+0 group (70% high-fat diet animal).

Microcomputed tomography (micro-CT) scans were performed to assess the volume of fatty tissue (VAT) in the liver. A representative result from each group was randomly selected, as shown in Figure 7.

The contrast-enhanced CT scans showed no significant difference in liver fat content at the end of the experiment between the different fatty liver groups.

#### 3.1.4. Blood Test

The possible changes in liver function were determined by taking blood samples from the experimental animals using standard laboratory methods. The results obtained from a representative sample of each fatty liver group are presented in Table 3.

The elevated levels of GOT, GPT and γ-GT are the most sensitive markers of possible liver damage. When the 3-month dietary period ended, the serological results showed no elevated liver enzyme levels even in the group with moderate steatosis (SAF 2+0+0). However, this group showed relatively higher levels of glucose, cholesterol and triglycerides compared to the others.

#### 3.1.5. Histological Analysis

**Figure 8:**
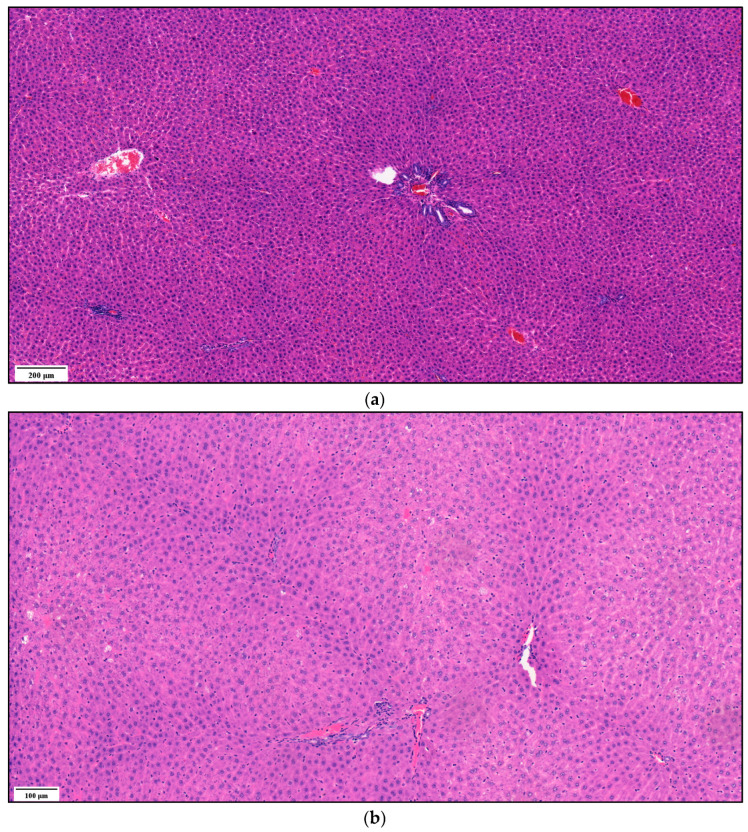

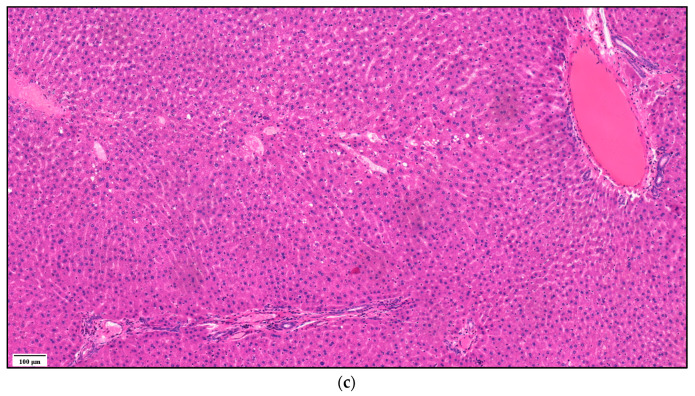
Histological features of liver tissue of Wistar rats with different SAF scales: (**a**) control group animal with 0+0+0 SAF value, (**b**) high-fat dietary group animal with 1+0+0 SAF value and (**c**) high-fat dietary group animal with 2+0+0 SAF value. Representative images of hematoxylin and eosin staining of liver tissue.

Representative rat liver specimens from the three groups of steatosis are shown in Figure 8. Figure 8a shows the histological analysis of a rat liver without steatosis, inflammation or fibrosis. It shows normal hepatocytes arranged in cords and obvious sinusoids, indicating that there are no pathological lesions. In Figure 8b, mild microvesicular steatosis (i.e., the accumulation of small fat droplets) can be seen in the cytosol of the hepatocyte. Figure 8c shows moderate steatosis.

### 3.2. Clinical Study

The clinical BIS measurements were performed in Department of Surgery of Clinical Centre University of Pécs, involving patients presented in Section 2.3.1. Patients underwent BIS measurement one day before surgery and liver tissue sampling during surgery.

#### 3.2.1. BIS Measurement

**Figure 9:**
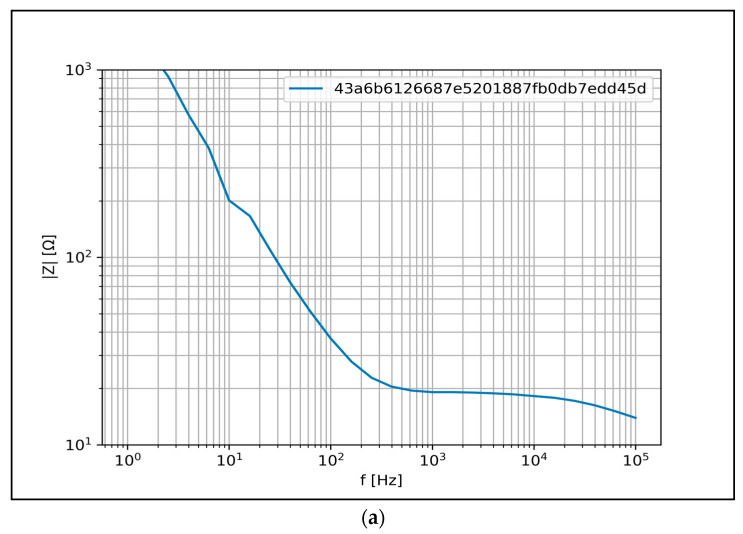

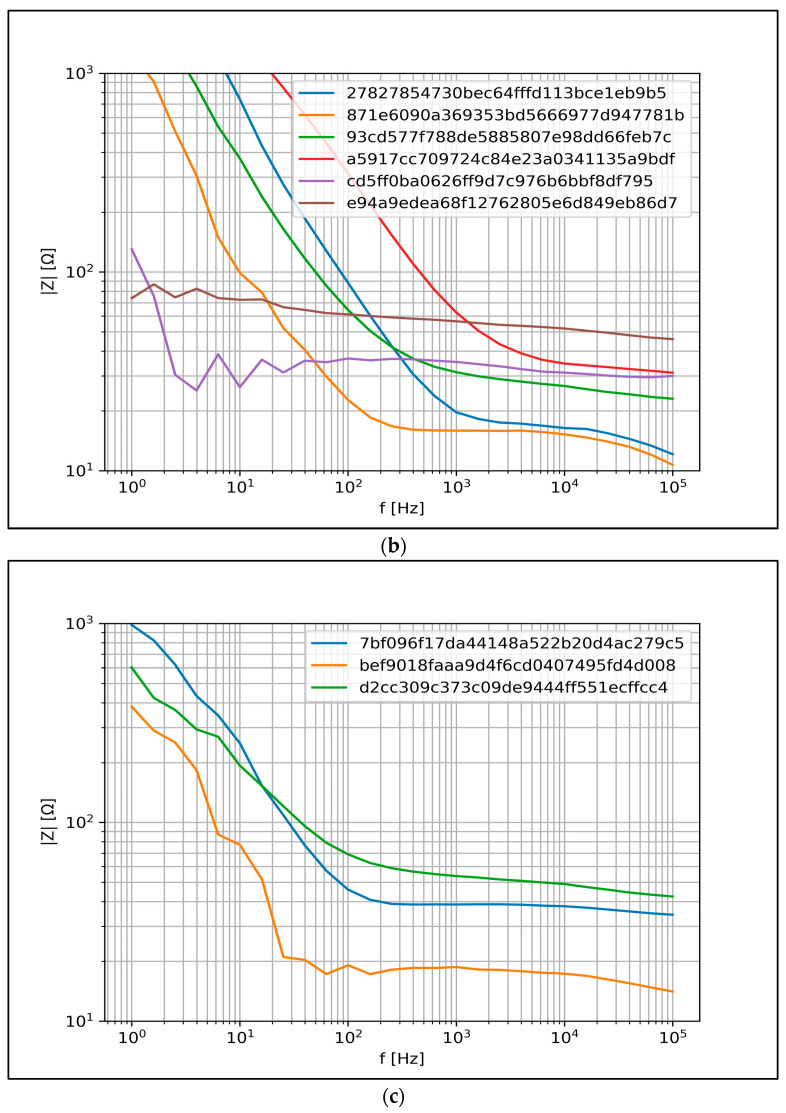
Typical BIS results in patients with different SAF scales: (**a**) 0+0+0 SAF value, (**b**) 1+0+0 SAF value and (**c**) 2+0+0 SAF value. Each curve represents a single human BIS measurement result, using different colors to distinguish patients from each other within the graph. To avoid patient identification, a code of letters and numbers was generated during the evaluation for each patient.

In presenting the clinical results, the spectra shown in Figure 9 are highlighted. Each curve represents human BIS measurement results that can be related to a certain SAF score. In each figure, the spectra are formed by the median of the five previously measured BIS values. To avoid the identification of the patient, a code of letters and numbers was generated instead of presenting personal data.

The purpose of examining BIS curves associated with different histological results is to demonstrate the variability of human BIS measurements. This paper focuses exclusively on the increasing effect of the degree of steatosis (S) while eliminating the changes caused by other values of the SAF score, namely activity (A) and fibrosis (F).

Hepatic steatosis is graded on a four-point scale (0 to 3). It only accounts for macro- and/or mediovascular steatosis and scores the percentage of steatotic vacuoles in hepatocytes. A normal liver (grade 0) has less than 5% of hepatocytes containing fat, while grade 1 steatosis indicates less than 33% of steatotic hepatocytes. In grade 2 and 3 steatosis, the fatty tissue is represented in at least 33% and 66% of the hepatocytes.

Figure 9a shows the BIS curve of a patient with SAF score results of 0+0+0. This means that all the values of steatosis, activity and fibrosis were zero based on the histological sampling results. The curve can be considered as a basis for further comparison. The curve shows a monotonically decreasing trend (“L” shape), with a definite breakpoint around 500 Hz, where the impedance value is up to 20 ohms. After the breakpoint, the downward trend changes to a short plateau phase up to 1000 Hz, followed by a downward tendency again.

Figure 9b shows the BIS spectra of patients with SAF scores of 1+0+0, which means that less than 33% of hepatocytes contain lipid droplets. The shape of the curves in this group also shows a monotonically decreasing trend with a pronounced breakpoint. The location of the breaking points varies from 200 Hz to 1000 Hz. The curves marked with yellow and blue colors have the common characteristic of a more sharply decreasing trend up to the break point (200/1000 Hz), followed by a less pronounced decrease up to the next breaking point (1000/2000 Hz) and finally a subsequent downward phase. The downward trend of the green and red curves is less marked compared to the yellow and blue curves; however, the breakpoint is shifted to higher frequencies of 500 Hz and 5000 Hz. The impedance values measured at the breakpoint also show higher values for the green and red curves (30 and 50 ohm), than for the yellow and blue curves (about 20 ohm). Compared to the yellow and blue curves, the green and red curves pass through a continuous low decreasing phase after the breaking points.

Figure 9c presents BIS spectra of patients with a 2+0+0 SAF result, indicating a moderate degree of steatosis. All three curves present a characteristic downward trending “L” shape with a distinct breakpoint around 100 Hz. The breakpoint above 100 Hz is followed by a plateau phase in each case. The impedance values recorded at 1 Hz remain overwhelmingly below 1000 Ohms. The yellow, blue and green curves show impedance values of approximately 20, 40 and 50 ohm during the plateau phase.

#### 3.2.2. Statistical Data

Table 4 summarizes the physical data of patients. Data were expressed as the mean and standard deviation of the age, weight, waist circumference and BMI. The first outstanding difference can be seen in the number of patients in the groups. Group SAF 0+0+0 had the lowest number of elements, while group SAF 1+0+0 was the most represented group. The mean age of the sample was 51 ± 11.5 years, and most of the participants (80%) were female. The body weight and waist circumference of patients shows an increasing trend from group SAF 0+0+0 to group SAF 2+0+0. The mean BMI was 29.9 ± 5.0 kg/m^2^. By examining each group individually, the first group of participants was found to be in the normal category, the second group showed overweight and the third group had a mild obesity score. In terms of BMI values, the 2+0+0 group showed the largest variance.

#### 3.2.3. Histological Analysis

**Figure 10:**
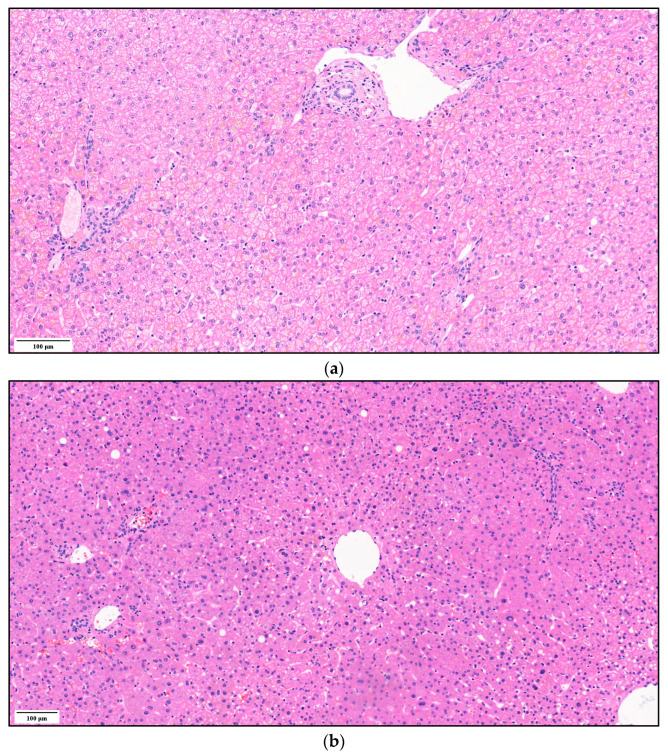

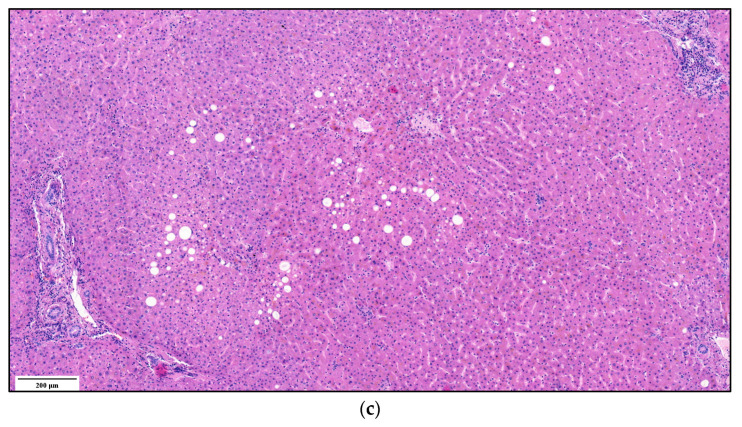
Histological features of liver tissue of clinical patients with different SAF scales: (**a**) patients with 0+0+0 SAF value, (**b**) patients with 1+0+0 SAF value and (**c**) patients with 2+0+0 SAF value. Representative images of hematoxylin and eosin staining of liver tissue.

Figure 10 illustrates the typical liver samples from the three groups of steatosis (SAF 0+0+0, 1+0+0, 2+0+0). Figure 10a shows normal liver structure with no histological evidence of hepatic steatosis. Figure 10b represents mild steatosis, including scattered fat droplet accumulations in liver hepatocytes. In Figure 10c, a moderate steatosis result is presented, with many fat droplets in the hepatocytes. The lipid vacuole characteristic of macrovesicular steatosis can be observed in the case of steatosis grade 1 (Figure 10b), but mainly in grade 2 (Figure 10c). They almost completely fill the entire hepatocyte, thereby squeezing the cell nucleus to the side. Extreme cases can also be observed in Figure 10c, where the cells appear to entirely comprise fat cells.

## 4. Discussion

This paper presents a new type of BIS-based measurement procedure developed to be suitable for early detection of NAFLD. To confirm this assumption, various levels of biological measurements (small laboratory animal experiment and clinical model) were designed. The bioimpedance data were collected using the same measurement principle for both levels of testing. The results presented in this paper can be interpreted as a continuation of previously started experiments [40].

Despite the relatively low number of elements, a similar tendency to the previously presented results was observed in the clinical results. The evaluation of BIS data and histological samples from the clinical trial found that there is a similarity between the curves in the same groups based on SAF score results. To present these groups, the BIS curves of the patients were presented with the characteristics specific for each group in Figure 9. The BIS data were analyzed visually by comparing the curves within the groups and also against other groups. The comparison of each group showed a steadily decreasing tendency in BIS slope below 100 Hz from the first to the last group. The impedance values at 100 kHz are increasingly higher in the control (SAF 0+0+0) group member. The BIS curves also represent a transition between the different groups. These BIS data indicate that further subgroups could be formed within each group.

The clustering observed in the human BIS results was also demonstrated by examining the impedance data from the small animal model experiments (Figure 6). Animals fed with artificial tube feeding or oral feeding with 70% and 35% high-fat diets for 3 months showed similar results to those observed in human studies. Based on the obtained impedance values, the same three groups of fatty liver could be generated as in the human studies (Figure 6). Comparing these individual groups also showed a decrease in the slope of the curve between the impedance values of each group, which was also seen in human measurements in Figure 9. As in the human study results, differences within groups can be discovered in this case of the animal model. Part of the SAF group 1+0+0 (Figure 6b) shows similarities with the curve characteristic of group 0+0+0 (Figure 6a), while the other part of the group shows values more characteristic of group 2+0+0 (Figure 6c).

Among the used imaging procedure for the detection of NAFLD, contrast CT scans and the standard serological liver function tests from blood samples were also performed in the case of small animal model experiment. The high-fat model reproduced the pathogenic factors related to the disease as the accumulation of lipid in the liver (Figure 8). In the case of the CT scans and blood tests, it could be concluded that no interpretable relationship was found between the CT (Figure 7) and liver function serological test (Table 3) evaluation of liver steatosis included in the animal model. This can be explained by the fact that liver enzymes can be intermittently normal at any histological stage of NAFLD; therefore, the presence of normal or near-normal aminotransferase levels does not exclude significant liver damage [43]. In contrast, common characteristics between BIS curves are classified into distinct groups based on the histological results.

This study includes some limitations. First, the clinical results are based on a small sample of a relatively healthy, middle-aged population; therefore, the generalization of the results to other populations may be limited. The number of patients was relatively small, mainly due to the fact that some patients showed not only simple steatosis, but also inflammation, or even fibrotic conditions, making it difficult to compare the different groups; therefore, these data were excluded. In addition, individual differences, such as body weight, waist circumference, hydration status, etc., were not taken into account in the assessment of the results. Finally, another limitation of this study is the limited correlation with imaging and liver function tests; hence, the sensitivity and specificity of the developed BIS method was not clearly compared to other generally used imaging and serological tests. This might be due to the limited selectivity and sensitivity of these techniques for the detection of simple steatosis, as discussed above [1,19].

The presented results are the raw data from a study that is still ongoing; therefore, a more detailed analysis will be needed in the future to interpret these results more comprehensively.

Further research will focus on improving and standardizing the proposed method, increasing the number of patients enrolled and including morbidly obese patients awaiting gastric bypass surgery and patients with peritoneal, renal, and other abdominal liver carcinomas. In vitro studies are also intended to be carried out.

## 5. Conclusions

In this paper, a novel noninvasive BIS-based method for the detection of hepatic steatosis was presented. It was observed that, both in humans and animal models, this method may be suitable for the staging of steatosis as well as for the early detection of the disease, either as part of a screening procedure or in a clinical setting. A more in-depth analysis is warranted to obtain complete understanding of the sensitivity and specificity of the new BIS method.

## Figures and Tables

**Table 1:** SAF score staging of steatosis according to the amount of liver fat.

Stage	Interpretation
S0	No steatosis (<5%)
S1	Mild steatosis (5–33%)
S2	Moderate steatosis (34–66%)
S3	Marked steatosis (>67%)

**Table 2:** Physical characteristics of Wistar rats included in the small animal experiment.

	All Animals	SAF 0+0+0	SAF 1+0+0	SAF 2+0+0
*n* (%)	60	23 (38%)	26 (43%)	11 (19%)
Day 1 body weight (g) (mean ± SD)	305.3 ± 50.4	308.4 ± 30.4	302.6 ± 49.6	304.9 ± 32.6
Day 90 body weight (g) (mean ± SD)	491.5 ± 79.8	391.8 ± 32.4	508.7 ± 72.9	534.7 ± 69.4
Liver weight (g) (mean ± SD)	14.4 ± 3.1	10.2 ± 0.9	14.7 ± 1.7	17.3 ± 3.2
Abdominal circumference (cm) (mean ± SD)	18.8 ± 2.3	16.9 ± 0.8	18.9 ± 2.1	20.2 ± 2.7

**Table 3:** Examples of rat’s liver function serological blood sample results.

Animal Number	AST/GOT (U/L)	ALT/GPT (U/L)	GGT/γGT (U/L)	Chol (mmol/L)	Trigl. (mmol/L)	HDL (mmol/L)	LDL (mmol/L)	Glucose (mmol/L)	Uric Acid (µmol/L)
20 (SAF score: 0)	377	79	0	1.73	1.27	1.05	0.32	6.68	64
4 (SAF score: 1)	503	66	0	1.97	0.97	1.2	0.48	5.7	86
10 (SAF score: 2)	199	47	0	2.11	2.82	1.03	0.62	9.79	49

**Table 4:** Physical characteristics of patients included in the clinical trial.

Characteristic/Group	All Patients	SAF 0+0+0 (*n* = 4)	SAF 1+0+0 (*n* = 15)	SAF 2+0+0 (*n* = 7)
Female (*n* (%))	21 (80%)	3 (75%)	11 (73%)	7 (100%)
Male (*n* (%))	5 (20%)	1 (25%)	4 (27%)	0 (0%)
Age (mean ± SD) years	51 ± 13.6	52 ± 12.1	50 ± 13.2	53 ± 16.9
Weight (mean ± SD) kg	84.6 ± 19.4	64.0 ± 7.1	88.0 ± 16.0	88.3 ± 25.5
Waist circumference (mean ± SD) cm	98.3 ± 13.6	87.0 ± 8.1	98.6 ± 13.4	104.3 ± 13.9
BMI (mean ± SD) kg/m^2^	29.9 ± 7.1	24 ± 2.6	30 ± 4.9	34 ± 9.9

## Data Availability

The data presented in this study are available on request from the corresponding author. The data are not publicly available due to privacy restrictions.
